# Intelligent Health Assessment of Aviation Bearing Based on Deep Transfer Graph Convolutional Networks under Large Speed Fluctuations

**DOI:** 10.3390/s23094379

**Published:** 2023-04-28

**Authors:** Xiaoli Zhao, Xingjun Zhu, Jianyong Yao, Wenxiang Deng, Yudong Cao, Peng Ding, Minping Jia, Haidong Shao

**Affiliations:** 1 School of Mechanical Engineering, Nanjing University of Science and Technology, Nanjing 210094, China; xlzhao@njust.edu.cn (X.Z.); zhuxj@njust.edu.cn (X.Z.);; 2 State Key Laboratory of Fluid Power and Mechatronic Systems, Zhejiang University, Hangzhou 310027, China; 3 School of Mechanical Engineering, Southeast University, Nanjing 211189, Chinampjia@seu.edu.cn (M.J.); 4 College of Mechanical and Vehicle Engineering, Hunan University, Changsha 410082, China

**Keywords:** aviation bearings, intelligent health assessment, large speed fluctuations, graph convolutional network (GCN), transfer learning

## Abstract

As a critical support and fixed component of aero engines, electro-hydrostatic actuators, and other equipment, the operation of aviation bearings is often subject to high speed, high-temperature rise, large load, and other continuous complex fluctuation conditions, which makes their health assessment tasks more difficult. To solve this problem, an intelligent health assessment method based on a new Deep Transfer Graph Convolutional Network (DTGCN) is proposed for aviation bearings under large speed fluctuation conditions. First, a new DTGCN algorithm is designed, which mainly uses the domain adaptation mechanism to enhance the performance of Graph Convolutional Network (GCN) and the generalization performance of transfer properties. Specifically, order spectrum analysis is employed to resample the vibration signals of aviation bearings and transform them into order spectral signals. Then, the trained 1dGCN is used as the feature extractor, and the designed Dynamic Multiple Kernel Maximum Mean Discrepancy (DMKMMD) is calculated to match the difference in edge distribution. Finally, the aligned features are fed into the softmax classifier for intelligent health assessment. The effectiveness of the proposed diagnostic algorithm and method are validated by using aviation bearing fault data set under large speed fluctuation conditions.

## 1. Introduction

Currently, high-speed rotating equipment represented by aero engines and aero-hydrostatic actuators is widely used in industry, defense, and military industries [1,2,3]. Specifically, aviation bearings and others are core components of the rotating equipment drive system. Its working environment is exceptionally harsh and complex, with frequent high speeds, high-temperature rises, large loads, and other continuously fluctuating conditions. Once the aviation bearing failure or damage directly affects the safety of aviation equipment, the light will lead to an abnormal increase in vibration or noise signals. Heavy will lead to irreversible catastrophic accidents [4,5]. Accordingly, it is necessary to carry out effective condition monitoring and fault diagnosis for bearings and other core components of equipment under complex operating conditions (such as variable conditions, speed fluctuation conditions, etc.) [4,5,6].

Specifically, the operating speed and load of aviation bearings are determined by the cruising speed and maneuvering action. The working condition of speed fluctuation is almost everywhere. The problems associated with intelligent health assessment of aviation bearings under large speed fluctuation conditions are illustrated below [7,8]: (1) The frequency domain signals of bearing faults under large speed fluctuation will generate frequency shift and amplitude change problems, which make the traditional signal processing methods unable to make accurate distinctions. (2) The time interval fluctuations between two adjacent shocks caused by speed changes. (3) Significant differences exist in the distribution between signals or samples. (4) The working environment is more complex, with many excitation sources, complex transmission paths, and more severe noise distribution. Namely, all these problems will make intelligent health assessment of aviation bearings more arduous.

For traditional aviation bearing fluctuating vibration signals, the main processing methods are based on two modes such as order-based analysis and time-frequency-based analysis [8,9,10]. Strictly speaking, the traditional spectral analysis method is employed to conduct discrete Fourier analysis on the vibration signal to obtain the main frequency components of the signal rather than the frequency change law with time. Therefore, the Fourier transform can only analyze the smooth signal if the spectral analysis interprets the vibration signal of the speed fluctuation. “Frequency blurring” and other phenomena are bound to occur. Specifically, short-time Fourier transforms (STFT), Gabor transforms, Wigner-Ville distribution (WVD), continuous wavelet transform (CWT), order tracking, and other methods can be employed to analyze non-stationary vibration signals [8,9,10]. The vibration of rotating machinery is often related to the rotational speed. Its operating state can be identified by the interrelationship between the order frequency components of the vibration signal proportional to the rotational speed. Therefore, order ratio analysis is more advantageous to represent the rotational speed-dependent vibrations well for monitoring the state characteristics of rotating machinery and practical health assessment [10].

Compared with the constant operating conditions, the forces on aviation bearings under fluctuating speed conditions are more variable and prone to failure. The mapping relationship between their signal signs and failure mechanisms becomes more complex. Therefore, studying bearing fault feature extraction and diagnosis under the large speed fluctuation condition is of practical theoretical and engineering value. Zhao, et al. [10], pointed out that the dynamic signals of mechanical equipment under variable speed will have non-smooth features such as frequency modulation, amplitude modulation, and modulation, which are often coupled with each other, making the fault feature extraction very difficult. Zhu, et al. [11], studied the transient shock response pattern of bearing faults under variable speed based on signal sparse representation theory, A sparse representation-based fault feature extraction method for variable speed bearings was proposed.

In summary, although the above-mentioned feature extraction methods have progressed and achieved the accuracy requirements for signal analysis under severe speed or load changes and random fluctuations, they still need to benefit from solving the high-precision time-frequency representation of arbitrary targets perfectly. Accordingly, the research on steady-state and relocatable fault feature extraction methods still needs to be explored and further decoded.

Deep learning (DL), one of the most advanced data and information processing tools, is used to enhance the accuracy of classification or prediction by multi-layer feature extraction [12,13]. Accordingly, scholars have researched intelligent fault diagnosis methods based on deep learning [13,14]. Miao, et al. [15], first used the AMESim simulation platform and experimental platform to simulate the fault of the aero-electro hydrostatic actuation system, then constructed an intelligent fault diagnosis model based on a deep convolutional neural network. Shao, et al. [13], designed a new fault diagnosis framework based on Dual-Threshold Attention-Guided Gan and limited infrared thermal images for rotating machinery. Zhao, et al. [1], developed an intelligent fault diagnosis method incorporating adaptive parametric rectifier linear units and deep residual networks for rotating machinery vibration signals that can vary significantly under different operating conditions. Due to the “high-dimensional and massive” state of the data measured by the aerospace equipment, the information from multiple sources from cross-coupling conditions, modes, and channels makes it difficult to guarantee the generalization of the diagnostic model. However, DL only performs nonlinear mapping of Euclidean space data, which is easy to ignore the interdependent correlation between data [13,14,15].

With the continuous in-depth research of DL models, many deep learning algorithms are combined with graph theory, where deep neural networks are injected with fresh blood [16,17,18,19]. Currently, the hot graph neural networks (GNN) focus more on the connection relationship of the data, and its goal is to establish specific network connections for the data stored in the graph domain to deal with the structural information of non-Euclidean spatial data. Owing to traditional neural networks are not translation invariant on non-Euclidean data structures (they cannot employ kernels of the same size for convolution). Still, this type of data is widely available in the real world. Graph Convolutional Networks (GCN) [17] came into being to handle such data, making it possible to convolve on irregular graph structures. Recently, algorithms such as Graph Attention Network (GAT) [18] and Graph Convolutional Auto-encoder (GCAE) [19] have been proposed, which have stronger data sparsity and reconfigurability. In Ref. [20], an intelligent fault diagnosis framework for multi-receiver field graph convolutional networks was offered for the existing DL algorithms that cannot mine the correlation information between signals. Zhao, et al. [21], proposed an intelligent fault diagnosis method for electro-mechanical systems based on a novel semi-supervised graph convolutional deep belief networks (SSGCDBN) algorithm.

However, the fluctuations in real operating conditions can produce a series of disturbances to the training of traditional machine learning (e.g., shallow learning, DL, GNN, etc.) models, which can be summarized as follows [4,20,21]: (1) It is challenging to obtain fault data during the service life of an aviation bearing, as fault data and label data are relatively scarce, but traditional models need a sufficient amount for model training. (2) The distribution of bearing measurement data varies greatly across conditions and component levels, which is challenging to ensure that the trained model can be adapted to other distributed data, etc. Accordingly, these factors are the bottlenecks that hinder the development of aviation-bearing fault diagnosis and intelligent maintenance.

To this end, transfer learning (TL) [22,23] provides a novel solution to the aforementioned problem by applying models and knowledge learned in the old domain (source domain) to the new domain (target domain) by exploiting the similarity between data, tasks, or models. Liao, et al. [22], first proposed a deep semi-supervised domain generalization network for fault diagnosis under rotating machinery variable speed for the problem that testing and training samples are not independently and identically distributed. Li, et al. [23], also established a deep adversarial multi-classifier optimization model based on cross-domain fault diagnosis to solve the domain transfer problem. However, the large fluctuation of fault conditions and multi-source parameter coupling of aviation bearings can result in a geometric structure relationship. Moreover, the non-Euclidean space correlation between their graph domains needs to be addressed. To solve the above-mentioned problems, deep graph transfer learning (DGTL) [22,23,24,25,26] is born, mainly by fusing the geometric structure relationship extraction performance of graph neural network and the knowledge transfer ability and strong generalization, which opens up a new research idea to solve the existing problems of aerospace equipment fault diagnosis.

To solve the problem of fault diagnosis difficulties arising from fluctuating conditions of aviation bearings, a new deep graph transfer learning algorithm is designed in this paper. Specifically, an intelligent health assessment method is proposed based on a deep transfer graph convolutional network (DTGCN) for aviation bearings under large fluctuating working conditions. Firstly, the proposed DTGCN algorithm uses a feature transfer learning mechanism to enhance GCN to have both solid geometric feature extractions in the graph domain and stronger generalization performance with more robust transfer characteristics. The aviation-bearing fault dataset under large speed fluctuation has validated the proposed diagnostic method and algorithm. Namely, the main contributions of this paper are included in the following three aspects:

(1) A deep transfer graph convolutional network (named DTGCN) algorithm is first proposed in this paper;
(2) Based on the proposed DTGCN algorithm, an intelligent health assessment method based on DTGCN algorithm is proposed for aviation bearing under large speed fluctuations;
(3) The intelligent health assessment method based on DTGCN can be validated using experimental data from aero engine bearing failure simulation.

The rest of this paper is mainly described as follows. Section 2 reviews a brief theory of GCN and MKMMD. Section 3 describes the proposed approach in detail. The validation and analysis of case studies are presented in Section 4. Finally, some conclusions are summarized in Section 5.

## 2. Preliminaries

The basics of graph convolutional network (GCN) and multiple kernel maximum mean discrepancy (MKMMD) [24] can be briefly introduced in this section to provide a research basis for the subsequent design of intelligent health assessment algorithm and method.

### 2.1. Graph Convolutional Network (GCN)

**Figure 1:**
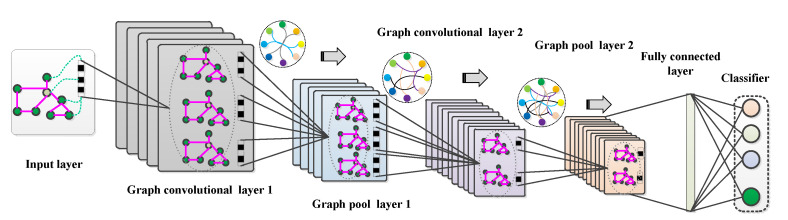
Schematic diagram of Graph Convolutional Network (GCN).

To effectively handle non-Euclidean spatial data, GCN [17] was created to perform convolution on irregular graph structures. The schematic diagram of GCN based on frequency domain convolution can be illustrated in Figure 1. The specific GCN implementation process is described as follows.

First, the spectral graph relationship with the Laplacian matrix as nodes and edges must be established. The graph can be represented as G (V, E, W), where V denotes the node in the graph, E denotes the edge between two nodes, and W denotes the edge weight between two vertices. In addition, the graph can be represented by a Laplace matrix as L = D − A, where D and A represent the degree matrix and adjacency matrix, respectively. Ultimately, the characteristic decomposition of the Laplacian matrix is defined as

(1) $$L=U\left(\begin{array}{l} \lambda_{1}\;\;\\ \;\ldots \;\\ \;\;\lambda_{n} \end{array}\right)U^{-1}=U\Lambda U^{-1}$$

where $U=\left[{\vec{u}}_{1},{\vec{u}}_{2},\cdot \cdot \cdot ,{\vec{u}}_{n}\right]$ is the unit eigenvector and Λ is a diagonal matrix consisting of the eigenvalues of the Laplacian matrix. Since U is an orthogonal matrix (i.e., UUT = E), its eigendecomposition can also be expressed as,

(2) $$L=U\left(\begin{array}{l} \lambda_{1}\;\;\\ \;\ldots \;\\ \;\;\lambda_{n} \end{array}\right)U^{T}=U\Lambda U^{T}$$

where is $U^{-1}=U^{T}$.

Based on the frequency domain convolution theory, the graph Fourier transform on the graph signal can be performed and convoluted in the spectral domain using the Fourier transform. Specifically, the graph Fourier transform operation is described as,

(3) $$F\left(\lambda_{l}\right)=\hat{f}(\lambda_{l})\sum_{i=1}^{N}f(i)u_{l}^{*}(i)$$

where *f* is the N-dimensional component of the graph, and *f*(*i*) corresponds to the graph nodes one by one. Denotes $u_{l}^{}(i)$ is the *i*-th element of the *l*-th feature vector and $u_{l}^{*}(i)$ is the conjugate vector $u_{l}^{}(i)$ of the representation.

The Fourier transform matrix form of the graph is $\hat{f}=U^{T}f$. Specifically, the Fourier transform has specific properties: $f\times g=F^{-1}\{F\text{\{}f\text{\}}\cdot F\text{\{}g\text{\}}\}$. Calculating the graph domain convolution is equivalent to Fourier domain multiplication, which first does the Fourier transform of the graph and convolution kernel after multiplication. Then, the Fourier inverse transforms back. The graph domain convolution can be obtained. The corresponding graph Fourier inverse transform is written as,

(4) $$f\left(i\right)=\sum_{i=1}^{N}\hat{f}(\lambda_{i})u_{l}(i).$$

According to the convolution theorem (i.e., the convolution of both functions *f*(*t*) and *g*(*t*) is the inverse of the product of the Fourier transforms of their functions), the process of convolution of the graph is rewritten as,

(5) $${\left(f*g\right)}_{G}=U\left(\begin{array}{l} g(\lambda_{1})\;\;\\ \;\ldots \;\\ \;\;g(\lambda_{n}) \end{array}\right)U^{T}f$$

where ${\left(f*g\right)}_{G}$ denotes the convolution of the functions *f*(*t*) and *g*(*t*), *U^T^f* denotes the Fourier transform, and g denotes the convolution kernel.

According to the properties of the Laplacian matrix, there is ${\left(f\times h\right)}_{G}=U(U^{T}(h)\cdot U^{T}(f))$, where ∘ is the Hadamard product, which represents the element-by-element product of two vectors of the same dimension at the corresponding positions. The output of the graph convolution network can be simply expressed as,

(6) $$y_{output}=\sigma (Ug_{\theta }(\lambda )U^{T}x)$$

More theoretical knowledge about GCN can be found in Ref. [17].

### 2.2. Multi Kernel-Maximum Mean Discrepancies (MKMMD)

MKMMD [24] is one of the most commonly used non-parametric methods to measure the difference in distribution between two domain datasets. Specifically, the feature representations in the source and target domains in transfer learning are mapped into the reproducing kernel Hilbert space (RKHS). Then the distance between the means of the two data types is calculated. The mean matching is calculated spatially to estimate the variability between edge distributions. Specifically, let *h^s^* and *h^t^* denote two types of deep features that fluctuate or cross-machine/cross-bearing, respectively, then MKMMD distance based on the two types of features is described as,

(7) $$\ell_{MK-\mathrm{MMD}}\left(h^{s},h^{t}\right)={\left\|\frac{1}{M_{s}}\sum_{i=1}^{M_{s}}\phi (h_{i}^{s})-\frac{1}{M_{t}}\sum_{i=1}^{M_{t}}\phi (h_{i}^{t})\right\|}_{H}^{2}$$

where ${\left\|\cdot \right\|}_{H}$ denotes the regenerated kernel Hilbert space and $\phi \left(\cdot \right)$ represents a series of characteristic mapping functions associated with the kernel mapping $k\left(h^{s},h^{t}\right)=\langle \phi (h^{s}),\phi (h^{t})\rangle$ for mapping the original variables to the regenerated kernel Hilbert space, $k_{l}\left(h^{s},h^{t}\right)$ defined as a convex combination of the l underlying kernels.

## 3. The Proposed Method

### 3.1. Problem Description

**Figure 2:**
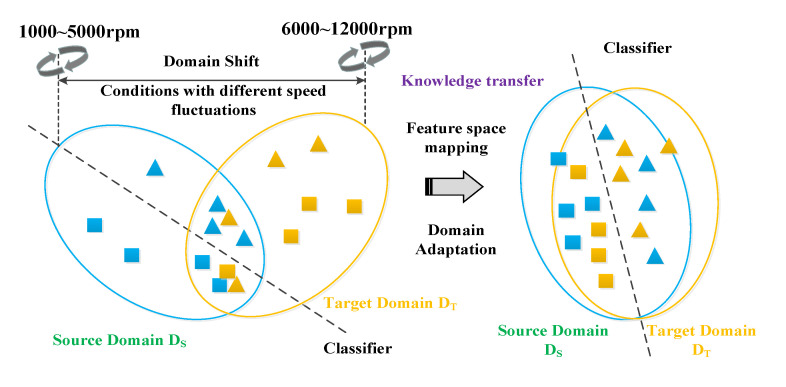
Problem description for domain adaptation based-intelligent health assessment.

Specifically, the domain adaptation problem and task for cross-bearing fault feature extraction can be described as follows [27,28]: First, assuming that a labeled source domain $D_{s}={\left\{x_{s}^{i},y_{s}^{i}\right\}}_{i=1}^{n}$ and an unlabeled target domain $D_{t}={\left\{x_{s}^{i}\right\}}_{i=1}^{m}$ are given. However, the edge and the conditional distribution are different, i.e., $P\left(x_{s}\right)\neq P\left(x_{t}\right)$ and $Q\left(y_{s},x_{s}\right)\neq Q\left(y_{t},x_{t}\right)$. Accordingly, the model trained based on the source domain cannot be used directly in the target domain. Accordingly, the source and target domains can be mapped to a shared feature space by constructing a mapping relationship *f*. The mapped feature distribution satisfies the requirement that the source domain distribution is approximately equal to the target domain distribution, which can be used for target domain classification, and other problems described in Figure 2.

### 3.2. The Constructed DTGCN Algorithm

**Figure 3:**
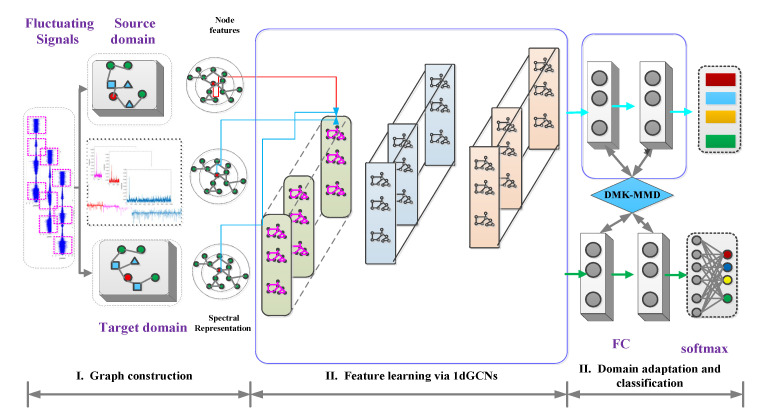
Schematic structure of the designed deep transfer graph convolutional network algorithm.

To implement domain adaptation fault feature extraction and intelligent health assessment under fluctuating operating conditions, the proposed DTGCN-based-intelligent health assessment is designed in this section. This algorithm mainly consists of three components: feature extractor G, label predictor P, and domain discriminator D. Specifically, the structure of the proposed DTGCN algorithm is schematically displayed in Figure 3. In addition, the proposed DTGCN algorithm mainly involves a graph construction phase, a 1dGCN feature learning phase, and a domain adaptation and classification phase, which are illustrated below.

#### 3.2.1. Graph Learning Construction Stage

Firstly, the multi-head attention mechanism and one-dimensional GCN are mainly used to construct the graph representation layer, graph attention layer, graph convolution, and graph pooling layer for multi-source signals and information fuse. The specific operation process is illustrated as follows.

**Figure 4:**
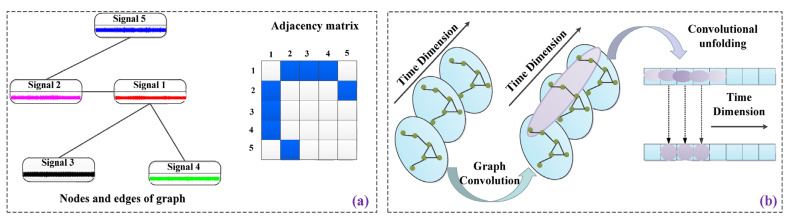
Graph representation learning core steps: (**a**) Sample relationship graph; (**b**) graph convolution process.

Graph representation layer: An undirected connected graph can be defined as $G=\left\{{\left(V_{i}\right)}_{i=1}^{n},{\left(E_{j}\right)}_{j=1}^{m},A\in R^{N\times N}\right\}$. $\left\{{\left(V_{i}\right)}_{i=1}^{n}\right\}$ represents all samples in the graph and the vertices that make up the graph. $\left\{{\left(E_{j}\right)}_{j=1}^{m}\right\}$ denotes all edges connecting the vertices. Based on this, the algorithmic steps are refined, and the core steps of graph representation learning are described in Figure 4.

#### 3.2.2. Feature Fusion and Extraction Stage

Graph attention layer: after realizing the signal-to-graph domain conversion, the graph attention layer inputs are both two sets of signals, which are randomly selected in the same domain with $V^{b}=\left\{v_{1}^{b},v_{2}^{b},\ldots ,v_{n}^{b}\left|v_{i}^{b}\in R^{L}\right.\right\}$ and $V^{c}=\left\{v_{1}^{c},v_{2}^{c},\ldots ,v_{n}^{c}\left|v_{i}^{c}\in R^{L}\right.\right\}$ corresponding to the adjacency matrix $A^{bc}\in R^{n\times n}$, respectively. *L* is the signal length. *A^bc^* is extracted by *F*(·) for each signal, and the Euclidean distance between two features is calculated as

(8) $$A^{bc}=\left[\begin{array}{l} {\left\|F\left(v_{1}^{b}\right),F\left(v_{1}^{c}\right)\right\|}_{2}\;{\left\|F\left(v_{1}^{b}\right),F\left(v_{2}^{c}\right)\right\|}_{2}\;\cdot \cdot \cdot {\left\|F\left(v_{1}^{b}\right),F\left(v_{n}^{c}\right)\right\|}_{2}\\ {\left\|F\left(v_{2}^{b}\right),F\left(v_{1}^{c}\right)\right\|}_{2}\;{\left\|F\left(v_{2}^{b}\right),F\left(v_{2}^{c}\right)\right\|}_{2}\;\cdot \cdot \cdot {\left\|F\left(v_{2}^{b}\right),F\left(v_{n}^{c}\right)\right\|}_{2}\\ \;:\;\cdot \cdot \cdot \\ {\left\|F\left(v_{n}^{b}\right),F\left(v_{1}^{c}\right)\right\|}_{2}\;{\left\|F\left(v_{n}^{b}\right),F\left(v_{2}^{c}\right)\right\|}_{2}\;\cdot \cdot \cdot {\left\|F\left(v_{n}^{b}\right),F\left(v_{n}^{c}\right)\right\|}_{2} \end{array}\right]$$

Each element of *A^bc^* represents the correlation between two signal samples. With the benefit of *A^bc^* revealing the correlation between *V^b^* and *V^c^* vertices, the graph attention layer efficiently fuses the information contained in *V^b^* and *V^c^*, and the attention matrix *E* can be calculated as

(9) $$E=Attention\left(V^{b}W^{b},V^{c}W^{c}\right)$$

The multi-head-attention (MAE) mechanism is introduced to filter irrelevant information. Essentially, MAE is a combination of multiple self-attentive structures using Scaled Dot Product Attention (SDPA) to compute the input vector sequence query *Q*, key *K*, and value *V* attention output, which can be expressed as

(10) $$SDPA\left(Q,K,V\right)=soft\max\left(\frac{QK^{T}}{\sqrt{d}}\right)V$$

where $Q=W^{q}e_{i}$, $K=W^{k}e_{i}$, $V=W^{v}e_{i}$, *d* is the network hidden layer size, i.e., the projection size. Accordingly, the input features in the MAE are projected to different subspaces by multiple self-attention operations, and then the set of multiple attention vectors are calculated as

(11) $$Mutilhead\left(Q,K,V\right)=Concat\left(head_{1},head_{2},\cdot \cdot \cdot ,head_{n}\right)$$

(12) $$head_{i}=Attention\left(QW_{i}^{Q},KW_{i}^{K},VW_{i}^{V}\right)$$

The multi-headed attention output is cascaded with a single-layer feedforward network, and the output is obtained. Then, each head attention output can be expressed as

(13) $$head_{i}=SDPA\left(QW_{i}^{Q},KW_{i}^{K},VW_{i}^{V}\right)$$

Equation (9) to Equation (13) were employed to weigh the combined form to form a multi-head attention matrix.

First, the graph structure of the multi-source signal at a specific moment is normalized $L=I_{N}-D^{-1/2}AD^{-1/2}\in R^{N\times N}$, where ***I****_N_* denotes the unit matrix, ***A*** denotes the adjacency matrix, and ***D***∈***R****^N^*^×*N*^ denotes the diagonal matrix ($D_{ii}\in \sum_{j}A_{ij}$). Since the Laplacian matrix ***L*** is a real symmetric matrix, an eigenvalue decomposition $L=U\Lambda U^{T}$ is performed on it, where ***U*** denotes the Fourier basis $L=U\Lambda U^{T}$ and the diagonal matrix $\Lambda =diag\left(\left[\lambda_{0},\ldots ,\lambda_{N-1}\right]\right)\in R^{N\times N}$ is composed of eigenvalues. The graph signal *x* of Fourier transform can be expressed as $\hat{x}=U^{T}x$, where *x* is the graph signals from feature fusion. Since *U* is an orthogonal matrix, its Fourier inverse transform is $x=U\hat{x}$. Doing the convolution operator *g* and the graph Fourier transform of the graph signal *x* separately, multiplying the transform results, and then obtaining the graph convolution results by the graph Fourier inverse transform,

(14) $$g*x=U\left(U^{T}g\cdot U^{T}x\right)$$

Here, * specifically refers to the graph convolution operation. Therefore, Chebyshev polynomials are proposed for the approximate solution with guaranteed accuracy. Ultimately, the propagation rule of the convolutional layer in GCN is expressed as

(15) $$H^{\left(l+1\right)}=\sigma \left({\tilde{D}}^{-1/2}A{\tilde{D}}^{-1/2}H^{\left(l\right)}W^{\left(l\right)}\right)$$

where $\sigma \left(\cdot \right)$ is the activation function, H is the output, W is the weight matrix, and *l* is the number of layers. Finally, GCN is then formed.

#### 3.2.3. Domain Adaptation and Classification Stages

Accordingly, the feature extractor consists of three 1dGCN layers and one fully connected layer, and the propagation rules of the 1dGCN layer are described above. The corresponding domain discriminator *D* determines whether the data comes from the source or target domain, takes the learned features as input, and outputs the predicted domain labels *D*(*G*(*x*)).

Therefore, the domain adversarial learning strategy is introduced to minimize the training loss function of the feature extractor *G*, and the training loss function of the domain discriminator *D* is maximized. Through such a dynamic “game” process, the feature extractor *G* and the domain discriminator *D* are optimally trained. Finally, the label predictor *P* is further used to optimize and predict *G*(*x*). Assuming that the parameters of the feature extractor, label predictor, and domain discriminator are *θ_G_*, *θ_P_*, and *θ_D_*, respectively, the loss function in model optimization can be expressed as

(16) $$\begin{array}{l} L\left(\theta_{G},\theta_{D},\theta_{P}\right)=\frac{1}{ns}\sum_{x_{i}\in D_{s}}L_{p}\left(P(G(x_{i})),y_{i}\right)-\\ \frac{\lambda }{ns+nt}\sum_{x_{i}\in (D_{s}\cup D_{t})}L_{D}\left(D(G(x_{i})),d_{i}\right) \end{array}$$

where *L_p_* and *L_D_* are label predictor and domain discriminator, respectively. λ are the adjustment factors, and *d_i_* is the label of inputting samples. Finally, the label predictor parameters are updated by minimizing and maximizing the objective function to update the domain discriminator parameters.

For the distribution constraint of DMKMMD, when the edge distributions *D_f_* of two domain features are different, the dynamic distribution alignment ${\bar{D}}_{f}$ can be quantitatively evaluated ($D_{f}$ and $D_{f}^{c}$). ${\bar{D}}_{f}$ can be expressed as

(17) $${\bar{D}}_{f}=(1-\gamma )D_{f}(P_{s},P_{t})+\gamma D_{f}^{c}(P_{s},P_{t})$$

where the adaptive factors γ∈[0,1] are used for the alignment weights $D_{f}^{c}$ and $D_{f}$. The dynamic alignment distribution for the domain invariant mapping *f* can be further expressed as

(18) $$\begin{array}{l} {\bar{D}}_{f}=(1-\gamma ){\left\|\frac{1}{n}\sum_{i=1}^{n}f(z_{i})-\frac{1}{m}\sum_{j=n+1}^{n+m}f(z_{j})\right\|}_{H_{K}}^{2}\\ \;+\gamma {\left\|\frac{1}{n}\sum_{i=1}^{n}f(z_{i}^{c})-\frac{1}{m}\sum_{j=n+1}^{n+m}f(z_{j}^{c})\right\|}_{H_{K}}^{2}=tr\left(\tilde{G}M_{0}\right)+tr\left(\tilde{G}M_{c}\right) \end{array}$$

where tr denotes the operation of finding the matrix trace, and the optimal combination of multiple kernels makes the feature extraction have a more accurate and reasonable expression by setting *K* to be a convex combination of *U* different kernel functions, which can be further expressed as

(19) $$K:\left\{k=\sum_{u=1}^{U}a_{u}k_{u}\left|\sum_{u=1}^{U}a_{u}=1,au\geq 0\right.\right\}$$

The above *M_o_* and *M_c_* are the MMD matrices for conditional distribution adaptation and edge distribution adaptation, respectively, denoted as

(20) $$M_{0}=\left\{\begin{array}{l} \frac{1}{n^{2}}\left(z_{i},z_{j}\in D_{s}\right)\\ \frac{1}{m^{2}}\left(z_{i},z_{j}\in D_{t}\right)\\ -\frac{1}{mn}\;\left(\mathrm{otherwise}\right) \end{array}\right.$$

(21) $$M_{c}=\left\{\begin{array}{l} \frac{1}{n_{c}^{2}}\left(z_{i},z_{j}\in D_{s}^{\left(c\right)}\right)\\ \frac{1}{m_{c}^{2}}\left(z_{i},z_{j}\in D_{t}^{\left(c\right)}\right)\\ -\frac{1}{m_{c}n_{c}}\;\left(\left[\begin{array}{l} z_{i}\in D_{s}^{\left(c\right)},\;z_{j}\in D_{t}^{\left(c\right)}\\ z_{i}\in D_{t}^{\left(c\right)},z_{j}\in D_{s}^{\left(c\right)} \end{array}\right]\right)\\ 0\left(\mathrm{otherwise}\right) \end{array}\right.$$

The output of its hidden layer is used as the depth features, and the deep feature variability is measured and characterized under fluctuating working conditions. DMKMMD is employed for the feature distributions of the *s* source domains. The data are projected into the Hilbert space, where mean matching is computed over the space, enabling estimation of the variability between edge distributions and avoiding the computation of intermediate probability densities. Let *h^s^* and *h^t^* denote the two types of depth features of work fluctuations or cross-bearing, respectively. The DMKMMD distance based on the two types of features can be expressed as

(22) $$\ell_{DMK-\mathrm{MMD}}\left(h^{s},h^{t}\right)={\left\|\frac{1}{M_{s}}\sum_{i=1}^{M_{s}}\phi (h_{i}^{s})-\frac{1}{M_{t}}\sum_{i=1}^{M_{t}}\phi (h_{i}^{t})\right\|}_{H}^{2}$$

where ${\left\|\cdot \right\|}_{H}$ denotes the regenerative kernel Hilbert space and $\phi \left(\cdot \right)$ represents a series of feature mapping functions $k\left(h^{s},h^{t}\right)=\langle \phi (h^{s}),\phi (h^{t})\rangle$ associated with the kernel mapping, $k_{l}\left(h^{s},h^{t}\right)$ defined as a convex combination of *l* underlying kernels. Then the joint objective function with min-max optimization training strategy can be rewritten as

(23) $$\underset{C,Q,P}{\min}\underset{D}{\max}\ell_{GCNs}+\lambda_{1}\ell_{DMK\mathrm{MMD}}+\lambda_{2}\ell_{\mathrm{GAN}}$$

where $\ell_{MTGAE}$ is the GCN loss, $\ell_{DMKMMD}$ is the DMKMMD loss, and $\ell_{GAN}$ is the adversarial loss.

Accordingly, the training phase focuses on the dynamic learning and optimization of the diagnosis model by using domain data input to the built algorithm framework, and then it is employed by generating adversarial learning strategies and the distribution variability between DMKMMD-aligned domains, the joint objective function and the parameter optimization can be displayed as

(24) $$\begin{array}{l} {\hat{\theta }}_{E}=\arg\left\{\underset{\theta_{E}}{\min}L_{C}\left(\theta_{E},{\hat{\theta }}_{C}\right),\underset{\theta_{E}}{\max}L_{D}\left(\theta_{E},{\hat{\theta }}_{D}\right)\right\}\\ {\hat{\theta }}_{C}=\arg\underset{\theta_{C}}{\min}L_{C}\left({\hat{\theta }}_{E},\theta_{C}\right)\\ {\hat{\theta }}_{D}=\arg\underset{\theta_{D}}{\min}L_{D}\left({\hat{\theta }}_{E},\theta_{D}\right) \end{array}$$

### 3.3. The Proposed Intelligent Health Assessment Method for Aviation Bearings

The proposed health assessment method can be divided into two main steps: signal processing based on order ratio analysis and intelligent health assessment based on DTGCN algorithm.

#### 3.3.1. Signal Processing Based on Simultaneous Extraction Transform-Order Ratio Analysis

First, the time domain signal is low-pass filtered and downsampled to remove the high-frequency interference components. Then, the time-frequency spectrum of the down-sampled signal is obtained by using the synchronous extraction transform. Next, the instantaneous frequency curve *f*_Cs_(*k*) of the rotational frequency is obtained from the time-frequency spectrum by defining the peak search (*k* is the sampling point number), and then the instantaneous frequency curve is fitted by reconstructing the least-squares fitting method. Then, the instantaneous frequency curve *f_i_*(*t*) is obtained from the instantaneous frequency curve, and then the phase identification time scale *T_n_* is obtained from the instantaneous frequency curve. Finally, the original data are resampled by the phase identification time scale at equal angles to obtain the quasi-steady-state angular signal sequence *x*(*T_n_*).

(25) $$x(T_{n})=\sum_{m}x\left(m\Delta t_{s}\right)h_{s}(m\Delta t_{s}-T_{n})$$

where $\Delta t_{s}$ is the time domain sampling interval, $m\Delta t_{s}$ is the value around *T_n_*, and $h_{s}\left(t\right)$ is the different filter. Finally, the order ratio spectrum analysis is obtained.

#### 3.3.2. Feature Extraction and Intelligent Health Assessment Based on DTGCN Algorithm

The above transformed quasi-smooth spectral signals are firstly input into the DTGCN algorithm. The fault information under fluctuating operating conditions is represented through the graph learning layer. The shared high dimensions of the source and target domains are extracted using the graph attention, graph convolution, and graph pooling layers (i.e., the improved 1DGCN), respectively. Afterward, DMKMMD and domain adversarial learning mechanisms are employed. Finally, the training of the health assessment algorithm under fluctuating conditions is implemented by the label predictor and domain discriminator.

**Figure 5:**
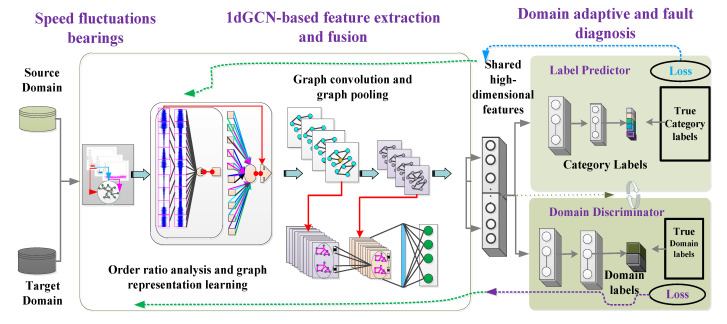
Flow chart of DTGCN-based intelligent health assessment method for aviation bearings.

Specifically, the flow chart of the DTGCN-based intelligent health assessment method for aviation bearings under large speed fluctuation conditions is described in Figure 5.

## 4. Validation and Analysis

### 4.1. Description for Aviation Bearing Fault Simulation Test Bench

**Figure 6:**
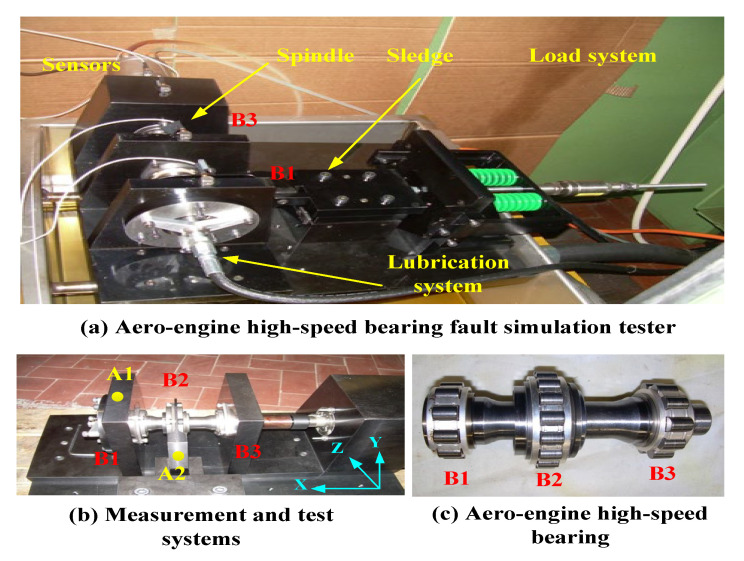
Aero engine high-speed bearing fault simulation test bench.

The data used in this section are from the aero engine high-speed bearing fault simulation test bench at the Department of Mechanical and Aerospace Engineering, Politecnico di Torino, Italy [29]. Specifically, the vibration acceleration data of aero bearings at different high speeds with other loads can be measured. A realistic view of the test rig is described in Figure 6. Accordingly, B1, B2, and B3 are three bearing supports. The triaxial vibration acceleration sensors are installed at A1 and A2, respectively. The vibration signal from the damaged bearing supports B1 and B2 can be measured and subjected to the maximum external load. The failure simulation process was roughly displayed as follows: First, the bearing was run briefly at 100 Hz rpm (6000 r/min) under no load, and after the correct installation, the magnitude of the external load was gradually changed, and the external load was slowly changed and increased in steps of 100 Hz to realize the modal simulation of speed fluctuation conditions. The rotational speed and external load of aviation bearing fault simulation are given in Table 1.

### 4.2. Construction of Aviation Bearing Dataset

**Figure 7:**
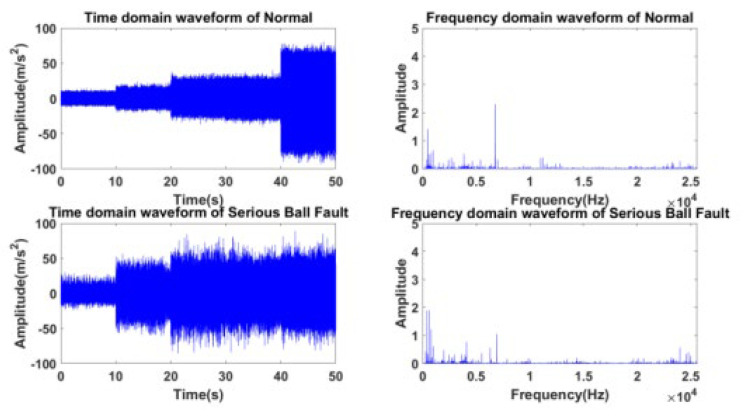
Time domain and frequency domain waveforms of H1 and H5 under rotational speed fluctuation.

To simulate the actual conditions of aero engine bearings operating at high speed and heavy load for a long time, vibration data under three loads (0 N, 1000 N, and 1400 N, respectively, A, B, and C data sets under fluctuating working conditions with speed fluctuation from 6000 rpm to 24,000 rpm) are selected to verify the effectiveness of the proposed method. Among them, the specific descriptions related to the typical experimental data set T1 are described in Table 2. Accordingly, the time domain and frequency domain waveform of specific speed fluctuations under the aviation bearing normal H1 and serious roller failure H5 are drawn in Figure 7.

### 4.3. Experimental Settings

To verify the effectiveness and generalization of the proposed method, cross-validation transfer tasks between different loads and rotational speeds are set. The specific transfer task numbers and rotational speed settings are described in Table 3. Accordingly, the number of samples in both source and target domains is set to 1000, and the sample length is 1024. FFT is the pre-processing data method to transform the original vibration signal into the frequency domain.

First, the main parameters of the constructed DTGCN algorithm model and the operating environment need to be briefly introduced and analyzed. Generally speaking, the number of network layers and the main parameters of the designed DTGCN are described in Table 3. In the following study, 10 trials of each experiment were performed for averaging to reduce the effect of randomness. The software tool used to run the fault diagnosis program was the PyCharm framework. Python was chosen as the programming language, and the programming framework for the deep learning algorithm was TensorFlow 1.2.0. The computer platform used for fault diagnosis was an i7-6700 with 8 cores and 8 GB of RAM, running in a Windows 64-bit operating system. The parameters of the weight initialization network and the model weights were updated using the Adam optimizer. A Dropout of 50% was implemented to reduce over-fitting.

### 4.4. Experimental Results

**Figure 8:**
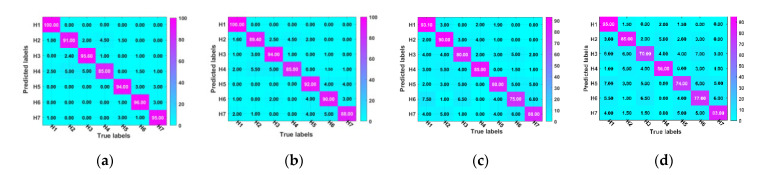
Diagnostic confusion matrix of the different methods at different tasks: (**a**) DTGCN (0.938, T1); (**b**) DTGCN (0.912, T2); (**c**) 1dGCN (0.833, T1); (**d**) 1dGCN (0.800, T2).

**Figure 9:**
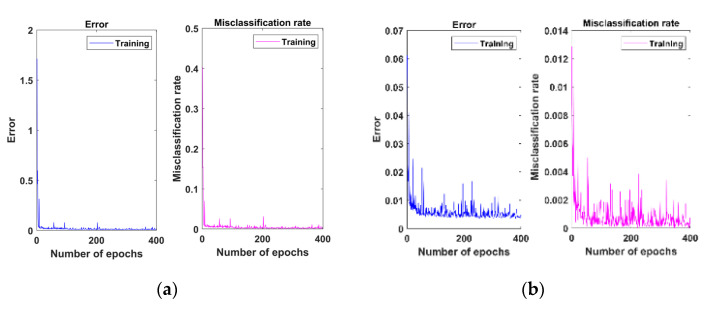
Training error curves of the proposed method and the standard algorithm: (**a**) DTGCN; (**b**) 1dGCN.

To further illustrate and analyze the proposed method’s effectiveness, the parameter-sharing mechanism selected 1dGCN as the benchmark comparison algorithm for DTGCN. Figure 8 shows the diagnostic confusion matrix of this diagnostic method based on DTGCNs and migrated 1dGCNs algorithm under different migration task conditions. The proposed method effectively classifies aerospace bearing faults under various operating conditions. Additionally, Figure 9 shows the training error curves under fluctuating operating conditions with different algorithms.

**Figure 10:**
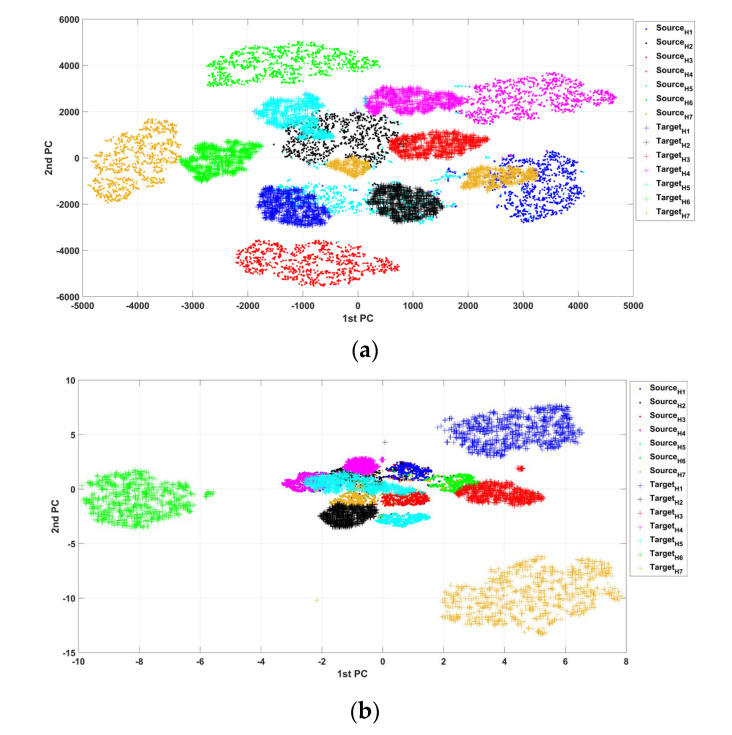
Visualization of low-dimensional embedded testing samples of different methods under fluctuating working conditions: (**a**) DTGCN; (**b**) 1dGCN.

To explore the characteristics of the feature maps in each layer of the deep neural network, the commonly used t-distributed stochastic neighbor embedding (t-SNE) [30,31,32,33,34,35,36] was used to reduce the high-dimensional features of the test samples. As shown in Figure 10a, compared with the ordinary 1DGCN model, the constructed DTGCN model reflects higher separability among testing samples under different health states, which verifies from the perspective of the distribution of test samples in the feature space the improvement of the diagnostic accuracy.

### 4.5. Comparison with Other Relevant Methods

CNN [6], GCN [17], Transfer Component Analysis (TCA) [30], JDA (Joint Distribution Adaptation) [31], Deep Adaptation Networks (Deep Adaptation Network (DAN) [32], and other DTGCN comparison methods were used to verify the effectiveness of the suggested method. Specifically, a preliminary normalization of the aforementioned datasets can be performed to improve the models’ diagnostic effectiveness, and we chose T1, T2, and T3 as specific training tasks multiple times as average results.

In addition, sensitivity and specificity were further introduced to evaluate the proposed model’s ability to predict different fault types. A positive and negative two-level problem was used as a better explanation. True positives (TP) are the number of correctly identified positive samples. False negatives (FN) are the number of samples incorrectly classified as positive. False Positive (FP) is the number of samples incorrectly classified as positive. True negative (TN) is the number of corrected and classified as negative samples.

**Figure 11:**
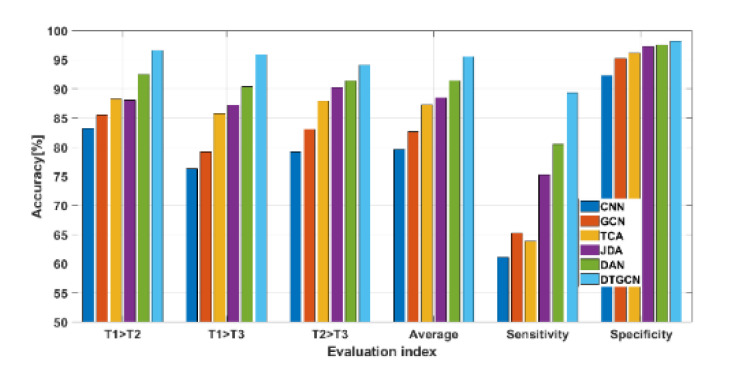
Comparison of diagnostic results of different diagnostic methods on the aerospace bearing dataset.

Generally, sensitivity and specificity can be defined as follows.

(26) $$\mathrm{Sensitivity}=\left(\mathrm{TP}/(\mathrm{TP}+\mathrm{FN})\right)$$

(27) $$\mathrm{Specificity}=\mathrm{TN}/\left(\mathrm{TN}+\mathrm{FP}\right)$$

where sensitivity measures the proportion of actual positives correctly identified, and specificity measures the ratio of real negatives correctly identified. Good models usually have high sensitivity and high specificity. Finally, the specific diagnostic results of the above-mentioned diagnostic models for the above three tasks are displayed in Figure 11. Accordingly, DTGCN achieves better migration diagnosis results in different migration tasks with an average accuracy of 95.57%. Owing DTGCN utilizes the nearest neighbor extraction performance of the geometric structure of 1dGCNs, which has a powerful transfer learning capability.

**Figure 12:**
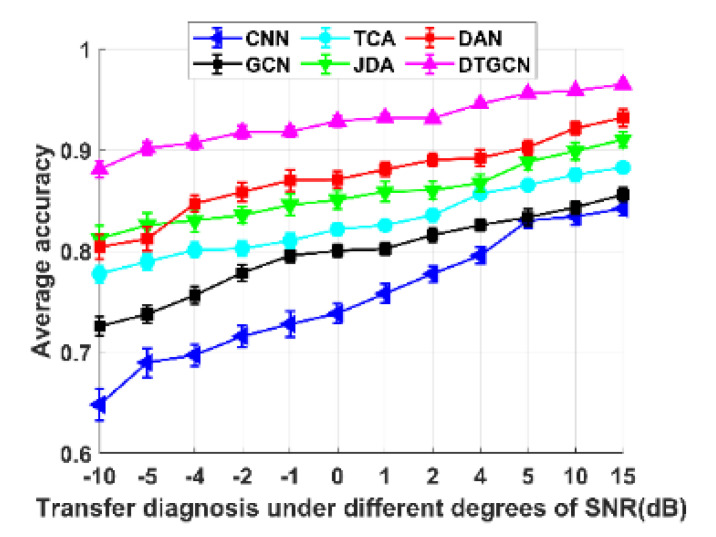
Average accuracy under different random noise.

To validate the superiority of the proposed method, the above-mentioned related algorithms were selected for comparison with the proposed DTGCN algorithm. In addition, it should be noted that the variable condition dataset utilized here is between 6000 and 24,000 rpm. The model parameters are mainly tuned several times according to the above experiments. T4, T5, and T6 are selected as migration tasks. The training and testing sample subsets are input to the above six types of fault diagnosis methods for diagnosis to obtain the diagnosis recognition accuracy after multiple averaging. To increase the difficulty of diagnosis and recognition, Gaussian white noise with different degrees of signal-to-noise ratio (SNR) is added to the original fluctuating working condition aviation-bearing fault data set to simulate the actual industrial fault diagnosis situation [30,31,32,33]. Meanwhile, 60% of the new data set after noise addition is randomly divided into training sample sets, and the remaining 40% is the testing sample set. The diagnostic recognition accuracy of the above six diagnostic methods with different degrees of random noise interference is displayed in Figure 12.

From Figure 12, it can be seen that the recognition rate of all diagnostic methods decreases with increasing random noise interference. Still, the overall noise resistance performance of the DTGCN model is stable. Some slight random noise interference before SNR = 5 has little effect on DTGCN. The traditional variable condition fault diagnosis methods (such as CNN and GCN) are more influenced by random noise, and the multi-scale fault diagnosis methods have a specific good effect on fluctuating conditions. In summary, the DTGCN algorithm based on the diagnosis method is more stable and noise-resistant than other traditional diagnosis frameworks.

## 5. Conclusions

In future work, extensive research and experiments will be conducted to improve the CNN model further and fuse data from multiple sensors to improve the diagnostic accuracy and robustness of the method. An intelligent health assessment method based on a deep transfer graph convolutional network is proposed to solve the poor diagnostic effect of aviation bearings under large speed fluctuation conditions. First, a novel DTGCN algorithm is designed, which uses an adaptive domain mechanism to enhance the convolutional graph network to have strong geometric feature extraction performance in the graph domain, which is a more robust generalization performance in transfer characteristics. The aviation-bearing fault data set under fluctuating conditions validate and advance the proposed diagnostic algorithm and method. Accordingly, the experimental results illustrated that the proposed method has higher diagnostic accuracy and robustness, which can eliminate the variability of health state sample distribution under large speed fluctuation conditions. Future work will continue to conduct in-depth research on the diagnosis of cross-machine and cross-part generalization and transfer of key aero engine components under extreme working conditions.

## Figures and Tables

**Table 1:** Test load and speed conditions.

Rated Load/N	0	1000	1400	1800
Rotating speed (r/min)	6000	6000	6000	6000
	12,000	12,000	12,000	12,000
	18,000	18,000	18,000	18,000
	24,000	24,000	24,000	/
	30,000	30,000	/	/

**Table 2:** Description of aviation bearing data set T1 (load = 0 N).

Labels	Damaged Areas	Diameter (um)	Rotational Speed (r/min)	Number of Samples
H1	No	No	6000~24,000	1000
H2	Inner ring	450	6000~24,000	1000
H3	Inner ring	250	6000~24,000	1000
H4	Inner ring	150	6000~24,000	1000
H5	Roller	450	6000~24,000	1000
H6	Roller	250	6000~24,000	1000
H7	Roller	150	6000~24,000	1000

**Table 3:** Different transfer task settings.

Source Domain Data Set	Transfer Tasks	
Training sample set A	A→B (T1)	A→C (T2)
Training sample set B	B→C (T3)	B→A (T4)
Training sample set C	C→A (T5)	C→B (T6)

## Data Availability

Not applicable.
